# UPLC–PDA‐ESI–QTOF–MS/MS and GC‐MS analysis of Iranian *Dracocephalum moldavica* L.

**DOI:** 10.1002/fsn3.2396

**Published:** 2021-06-14

**Authors:** Azin Fattahi, Abolfazl Shakeri, Zahra Tayarani‐Najaran, Mourad Kharbach, Karen Segers, Yvan Vander Heyden, Seyedeh Faezeh Taghizadeh, Hanieh Rahmani, Javad Asili

**Affiliations:** ^1^ Department of Pharmacognosy School of Pharmacy Mashhad University of Medical Sciences Mashhad Iran; ^2^ Department of Pharmacodynamics and Toxicology School of Pharmacy Mashhad University of Medical Sciences Mashhad Iran; ^3^ Department of Analytical Chemistry Applied Chemometrics and Molecular Modelling Center for Pharmaceutical Research (CePhaR) Vrije Universiteit Brussel (VUB) Brussels Belgium; ^4^ Biopharmaceutical and Toxicological Analysis Research Team Laboratory of Pharmacology and Toxicology Faculty of Medicine and Pharmacy University Mohammed V‐Rabat Morocco; ^5^ Department of Horticultural Sciences Ferdowsi University of Mashhad Mashhad Iran; ^6^ Pharmaceutical Research Center Pharmaceutical Technology Institute Mashhad University of Medical Sciences Mashhad Iran

**Keywords:** antioxidant activity, cytotoxic activity, *Dracocephalum moldavica*, essential oil, GC‐MS, UPLC‐MS

## Abstract

*Dracocephalum moldavica* L. is a significant component in the Iranian food basket. This study aimed to investigate the bioactive compounds and biological activities of different extracts obtained from *D. moldavica* aerial parts. From the aerial parts, a crude methanolic (MeOH) extract and its four sub‐fractions, that is, petroleum ether (Pet), ethyl acetate (EtOAc), n‐butanol (n‐BuOH), and aqueous (water) extracts were obtained. The total phenolic and flavonoid contents as well as the antioxidant and cytotoxic activities of the extracts were determined. Moreover, the phytochemical profiles of the essential oil (EO) and of those extracts with the highest antioxidant activity measured by GC/MS and UPLC–PDA‐ESI–QTOF–MS/MS. Results showed that the highest concentrations of phenols and flavonoids as well as the most potent antioxidant potential according to the DPPH method were determined in the EtOAc and MeOH extracts with IC_50_ values of 22.0 and 34.4 µg.ml^‐1^, respectively. Quantitative analysis of these extracts was subsequently performed by UPLC–PDA‐ESI–QTOF–MS/MS. Both extracts contained mainly rosmarinic acid, caffeic acid, and 2‐hydroxycinnamic acid, which may be responsible for their high antioxidant activity. Moreover, none of the extracts showed cytotoxic effects against MCF7, SW48, and a normal cell line of mouse embryonic fibroblast cells (NIH/3T3) in the tested concentrations (up to 400 μg.ml^‐1^). Additionally, GC‐MS analysis showed that oxygenated monoterpenes (55.4%) were the main constituents of the EO of *D. moldavica*.

## Graphical abstract

*Dracocephalum moldavica* is a significant component in the Iranian food basket. The total phenolic and flavonoid contents as well as antioxidant and cytotoxic activities of *D*. *moldavica* extracts were determined. In addition, the phytochemical profile of the essential oil (EO) using GC/MS and those extracts with the highest antioxidant activity by UPLC–DAD‐ESI–QTOF–MS/MS were measured.

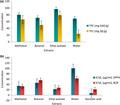

## 1 INTRODUCTION

The daily intake of sufficient vegetables has an important role in preventing several diseases (Barends et al., 2019). *D. moldavica* (Moldavian balm) is a common edible vegetable used daily for the preparation of many Iranian dishes. It belongs to the Lamiaceae family, is up to 80 cm tall, and is native to central Asia (Yousefzadeh et al., 2018). *D. moldavica* preparations are used in food and in pharmaceutical industries as food additive, tea, and herbal remedy. Traditionally, the plant is applied as analgesic, anticonvulsive, anti‐inflammatory, sedative, wound healing, and in the treatment of cardiovascular disorders (Yousefzadeh et al., 2013). In the Mexican traditional medicine, it is used for the treatment of nervous diseases (Martinez‐Vazquez et al., 2012), while in traditional Chinese medicine (TCM), it is mainly used in the treatment of liver disorders, headache, stomach problems, and congestion (Jiang et al., 2014). Furthermore, in TCM in a clinical trial the aqueous extract of *D. moldavica* was shown to be effective in the treatment of cardiovascular disease, asthma, fatigue insomnia, and neurasthenia (N. Yu et al., 2015).

Phytochemical investigations on the aerial parts of *D. moldavica* have demonstrated the presence of several bioactive compounds, including terpenoids, phenolic compounds (rosmarinic and caffeic acid derivatives), flavonoids (kaempferol, quercetin, esculetin, diosmetin, acacetin, apigenin, luteolin, cirsimaritin, salvigenin, santa flavone, agastachoside, and their glycosides), alkaloids, iridoids, and coumarins (Sultan et al., 2008; Yang et al., 2014; Zeng et al., 2010). Phenolic compounds, especially phenolic acid derivatives, such as rosmarinic and caffeic acids, were associated with the high antioxidant potential of *D. moldavica* (Weremczuk‐Jeżyna et al., 2013). Various analytical methods are developed for the identification and quantification of bioactive compounds in medicinal plants. However, in these samples, there are some limitations, including the complexity, the structural diversity, and the low content of bioactive compounds (Adnani et al., 2012). In this regard, the choice of an appropriate technique is important. The application of UPLC‐ESI‐MS in the identification of natural compounds has attracted much attention because of its high resolution for the separation of complicated samples, analysis speed, sensitivity, selectivity, specificity, and reduced solvent consumption (Chen et al., 2010). As it is a significant component in the Iranian food basket, *D. moldavica* was selected for this study. To the best of our knowledge, there is no comprehensive study on this edible vegetable plant. Therefore, for the comprehensive identification and quantification of the chemical composition of *D. moldavica*, UPLC–DAD‐ESI–QTOF–MS/MS was used as a powerful tool for the separation of low molecular weight and nonvolatile samples, and GC/MS for the separation of volatile and thermally stable compounds. As biological activities, we evaluated the antioxidant and cytotoxic abilities of different plant extracts. Our study established a new approach to explore comprehensively the chemical components of *D. moldavica* extracts using UPLC–PDA‐ESI–QTOF–MS/MS. The obtained results broaden our knowledge about the structural diversity of the components in Moldavian balm for a better understanding of the possible role of the constituents on biological properties as well as for further research in food and pharmaceutical issues.

## 2 MATERIAL AND METHODS

### 2.1 Plant material

*D. moldavica* was purchased from a local market in Mashhad city (Khorasan Razavi province, Northeastern of Iran) in September 2017. The plant material was identified by M. Souzani (Department of Pharmacognosy, Mashhad University of Medical Sciences) and a voucher specimen (10,169) was deposited in the herbarium of the Department of Pharmacognosy, Mashhad University of Medical Sciences.

### 2.2 Preparation of the extracts

The aerial parts were washed with tap water and dried. For extraction of plant materials, all solvents were purchased from Dr. Mojallali Industrial Chemical Complex Co. 400 g dried material was powdered and macerated in methanol (analytical grade, 99.5%) for 24 hr (3 times, 1 L) at room temperature. The obtained extract was filtered using filter papers (Whatman^®^ No.1, Merck) and the organic solvent concentrated under a vacuum. Then, the entire extract was suspended in water (50 ml) and partitioned with Pet (200 ml), EtOAc (200 ml), and n‐BuOH (200 ml), successively. Afterward, the solvents were evaporated under reduced pressure to get the different sub‐fractions. To prepare the EO, the aerial parts of the plant were subjected to hydrodistillation (Clevenger‐type apparatus, Pyrexfan Co) for 3 hr. The obtained EO was dried over anhydrous sodium sulfate (Merck) and stored in the dark until further testing.

### 2.3 Total phenolic content (TPC)

The TPC was measured colorimetrically with a standard Folin–Ciocalteu method (Slinkard & Singleton, 1977). The extract (20 μl) was mixed with 1,160 μl distilled water and 100 μl Folin‐Ciocalteu reagent (Merck). After 5 min, 300 μl sodium bicarbonate (20%, Merck) solution was added to the mixture and kept at room temperature for 2 hr. Absorbance was read at 760 nm using a Biotech Plate Reader (BioTek Instruments). A calibration curve (5–80 μg/ml) was built with gallic acid (Sigma‐Aldrich), and TPC expressed in mg gallic acid per gram dried extract (mg GAE g^‐1^).

### 2.4 Total flavonoid content

The TFC was determined by the aluminum chloride colorimetric method (Chang et al., 2002). After mixing 500 μl extract with 100 μl aluminum chloride (10%, Merck)), 1,500 μl ethanol (95%), 100 μl potassium acetate (1 M, Merck), and 2,800 μl distilled water, the mixture was kept at room temperature for 30 min and the absorbance measured at 415 nm. The results were expressed as mg quercetin (≥95%, Merck) equivalents per gram dried extract (mg QE g^‐1^).

### 2.5 Antioxidant activity

#### 2.5.1 2,2‐Diphenyl‐1‐picrylhydrazyl (DPPH) radical scavenging

The free radical scavenging activity of extracts was tested by a DPPH test (Mensor et al., 2001). Briefly, 100 μl of different extract concentrations (12.5–400 μg.ml^‐1^) was added to 100 μl freshly prepared 0.1 mM DPPH (Merck) solution in methanol. After 30 min of reaction at 37℃ in the darkness, the absorbance of the sample was measured at 518 nm. Ascorbic acid was applied as positive control. In this method, DPPH (100 μl) + methanol (100 μl) are used as blank. The antioxidant capacity was then calculated using the following Equation (1):

(1) $$\text{AA}\%\;=\;\left[\left(A_{\text{blank}}‐{\;A}_{\text{sample}}\right)/A_{\text{blank}}\right]\;\times \;\text{1}00$$

#### 2.5.2 β‐carotene linoleic acid bleaching (BCB) assay

The BCB assay was conducted according to the standard method (Kulisic et al., 2004). In brief, *β*‐carotene (0.1 mg, ≥93%, Merck) was dissolved in 0.5 ml chloroform and mixed with 10 mg linoleic acid (≥99%, Merck) and 100 mg Tween‐40. Then, the chloroform was evaporated at 50℃, distilled water (25 ml) was added and the mixture sonicated for 1 min. An initial absorbance was recorded at 470 nm (time =0 min). Aliquots of the *β*‐carotene/linoleic acid solution (200 μl) were mixed with the prepared extracts (50 μl) and incubated at 50℃. The absorbance was measured at 470 nm after 120 min incubation. Antioxidant activity of the extracts was calculated by Equation (2):

(2) $$\%\;\text{Inhibition}=\;\left[\left(A_{A(\text{12}0)}‐A_{C(\text{12}0)}\right)\;/\;\left(A_{\left(C0\right)‐}A_{C(\text{12}0)}\right)\right]\;\times \;\text{1}00$$

where A_A(0)_ and A_A(120)_ are the absorbances of sample at times 0 and 120 min, while A_C(0)_ and A_C(120)_ are the absorbances of control after 0 and 120 min.

### 2.6 Cytotoxic activity

Human breast cancer cell line MCF7, colorectal cancer cell line SW48, and a normal cell line mouse embryonic fibroblast cells NIH 3T3 were provided by the National Cell Bank of Iran (Pasteur Institute). They were kept with 10% (v/v) fetal bovine serum (FBS) (Gibco), penicillin/streptomycin at 100 IU/ml and 2 mM L‐glutamine. Cultures were incubated with 5% CO_2_ in a humidified atmosphere at 37ºC. The cytotoxic effect of the prepared extracts was assessed using the AlamarBlue^®^ (BioSource Invitrogen) proliferation assay. Briefly, cells were seeded in 96‐well plates at a density of 1 × 10^4^. The cells were treated with different concentrations of extract (100 μl, 50–400 μg.ml^‐1^) after overnight growth. After 48 hr treatment, 20 μl AlamarBlue^®^ reagent was added to each well. After 2 to 4 hr, the absorbance at 600 nm was measured on a Biotech Plate Reader (BioTek Instruments). Doxorubicin (0.1, 0.5 and 2 μg.ml^‐1^) was chosen as a positive control. IC_50_ values were calculated from Boltzmann sigmoidal concentration–response curve nonlinear regression fitting models (Lyles et al., 2008).

### 2.7 Chemical profiles and phytochemical content

#### 2.7.1 Gas chromatography–mass spectrometry (GC‐MS)

The GC‐MS analyses were performed using a Agilent 5,975 apparatus with a HP‐5ms column (30 m × 0.25 mm i.d., 0.25 µm film thickness) interfaced with a quadruple mass detector and a computer equipped with Wiley 7n.l library. Instrumental conditions: oven temperature gradient: 50℃ during 5min, 50℃–250℃ at 3℃ /min and 250℃ during 10 min; injector temperature 250℃; injection volume, 1 µl; split ratio, 1:20; carrier gas, Helium at 1.0 ml/min; ionization potential, 70 eV; ionization current, 150 µA; ion source temperature, 280℃; mass range, 35–465 m*/z*. The constituents of the oils were identified by calculation of their retention indices under temperature programmed conditions for n‐alkanes (C_8_‐C_23_) and the oil on the HP‐5ms column (van Den Dool & Dec. Kratz, 1963). Identification of individual compounds was made by comparison of their mass spectra and retention indices (RI) with those of authentic samples and those given in the literature (Adams, 2007).

#### 2.7.2 Ultra‐performance liquid chromatography coupled with a photo diode array detector and electrospray ionization quadrupole time‐of‐flight mass spectrometry (UPLC‐PAD/ESI‐QTOF/MS)

An Acquity Ultra‐Performance Liquid Chromatograph (UPLC, Waters) coupled to a photo diode array detector (PDA, Waters) and an electrospray ionization quadrupole time‐of‐flight tandem mass‐spectrometer (ESI–QTOF/MS; Waters) was used. Chromatographic separation was done using an Acquity UPLC column (UPLC^®^ BEH C_18_, 100 mm × 2.1 mm, 1.7 μm, Waters). A binary mobile phase was used, mobile phase A (ultra‐pure water with 0.1% formic acid) and mobile phase B (acetonitrile with 0.1% formic acid). Formic acid and acetonitrile were UPLC‐MS grade from Actu, OSS, The Netherlands. A gradient separation was applied; 10% B, 0 min; 70% B, 30 min; 100% B, 33.33 min; 100% B, 38.33 min; 10% B, 41.67 min; 10% B, 50 min. The column temperature was maintained at 40℃, flow rate at 0.5 ml/min, wavelength range between 210 and 400 nm, and 10 µl sample was injected.

The ESI operating conditions for MS spectra acquisition in negative mode were as follows: capillary voltage, 2.6 kV; cone, 40 V; desolvation temperature 500℃; and source temperature, 150℃. The desolvation and cone gas flow rates were 0 and 1,000 L/h, respectively. Nitrogen (99.80% N28, Air Liquide, Auderghem, Belgium) was used for both desolvation and cone gas. Sample analysis was done independently in *MS^E^* acquisition (E is the collision energy) applying a full scan mode (50–1200 m*/z* range), in 1 s scan time. The precursor mass spectra acquisition was done in two continuous modes, a no collision energy mode, and a high collision energy (15–35 eV). Leucine enkephalin (Sigma‐Aldrich) was used as internal reference (LockSpray^™^) to calibrate the ESI source. The data were acquired by a MassLynx™ 4.1 software (Waters).

#### 2.7.3 Sample preparation

Plant extract, 4 mg, was dissolved in 2.0 ml water/methanol (1:1; v/v) and then mixed for 10 min. Then, the sample was filtered using a membrane filter (0.20‐μm) prior to injection.

#### 2.7.4 Identification and quantification of compounds

Compounds were identified and quantified in accordance to the retention times and mass spectral data (mass‐to‐charge (*m/z*), molecular peaks and their fragmentation) of the calibration standards. The analyte concentration was calculated using calibration curves of pure standards (Sigma‐Aldrich). Stock solution of each pure calibration standard (1 mg.ml^‐1^) was prepared in methanol, and dilutions were made at 6 levels (1, 5, 10, 25, 50, 100 μg.ml^‐1^) for the calibration curves. Results were expressed as μg.g^‐1^ pure extract. The quantification was done in duplicate.

## 3 RESULTS AND DISCUSSION

### 3.1 Essential oil composition

Seventy compounds, representing 99.6% of the EO of *D. moldavica,* were identified (Table 1). The main components were geranial (25.5%), estragole (16.0%), and geranyl acetate (15.2%). The majority of the compounds in the EO were oxygenated monoterpenes (55.4%). Golparvar et al., (2016) reported that *D. moldavica* EO collected from Kamu Mountain, Isfahan province, Iran, was dominated by geranyl acetate (36.62%), geraniol (24.3%), neral (16.2%), and geranial (11.2%). In a study by Yousefzadeha et al. (2018), geraniol, geranial, nerol, and geranyl acetate were the major constituents of the EO of *D. moldavica* collected from five habitats in the north‐west of Iran (Salmas, Urmia, Khoy, Maragheh, and Tabriz). Fallah et al., (2018) found that the major components of the EO of *D. moldavica* were geranyl acetate, neral, linalool acetate and geraniol. In another study (Fallah et al., 2018), geranial (29.0%–41.5%), geranyl acetate (24.7%–34.8%), and neral (21.9%–28.6%) were the main components of the EO of *D. moldavica*. Still different results were reported by some other researchers, who found that linalool (Hussein et al., 2006) and citral (Nikitina et al., 2008; Shuge et al., 2009) are the predominant components of *D. moldavica* EO. Such differences in EO composition are common and might be due to physiological variations as well as ecological and genetic factors, seasonal and climatic conditions, harvest period, and the distillation technique applied (Shakeri et al., 2019).

**TABLE 1:** Volatile components in the EO of *Deracocephalum moldavica*

No	Compound	RI^1^	Percentage (%)
1	Benzaldehyde	962	*t* ^2^
2	1‐octen‐3‐ol	982	0.1
3	6‐methyl‐5‐hepten‐2‐one	988	0.4
4	Myrcene	992	0.1
5	2E,4E‐heptadienal	1,011	*T*
6	*ρ*‐cymene	1,026	*T*
7	Limonene	1,030	0.1
8	*cis*‐ocimene	1,041	0.2
9	Benzene acetaldehyde	1,045	0.1
10	*trans*‐ocimene	1,052	0.1
11	Bergamal	1,058	0.1
12	*cis*‐linalool oxide	1,074	0.1
13	Terpinolene	1,089	0.1
14	*trans*‐linalool oxide	1,090	0.1
15	Linalool	1,101	1.3
16	1‐octen‐3‐yl acetate	1,115	0.1
17	Allo‐ocimene	1,133	0.1
18	*trans*‐chrysanthemal	1,154	0.1
19	Citronellal	1,156	0.1
20	Nerol oxide	1,159	0.1
**21**	**Methyl chavicol (estragole)**	**1,204**	**16.0**
22	4‐methylene isophorone	1,220	0.1
23	Nerol	1,232	0.3
24	Neral	1,254	9.7
25	Geraniol	1,258	0.5
**26**	**Geranial**	**1,280**	**25.5**
27	Unknown	1,302	0.2
28	Geranyl formate	1,306	0.4
29	Neryl acetate	1,365	1.2
30	α‐copaene	1,378	1.0
31	Nerolic acid	1,378	0.2
32	β‐bourbonene	1,389	0.3
**33**	**Geranyl acetate**	**1,390**	**15.2**
34	Geranic acid	1,406	0.2
35	Methyl eugenol	1,410	0.2
36	β‐caryophyllene	1,423	0.6
37	Unknown	1,430	0.1
38	β‐copaene	1,434	*T*
39	Dihydro‐β‐ionone	1,443	*T*
40	Aromadendrene	1,446	*T*
41	α‐humulene	1,457	0.2
42	E‐β‐farnesene	1,461	*T*
43	α‐amorphene	1,483	0.1
**44**	Germacrene D	1,486	0.3
45	E‐β‐ionone	1,490	0.5
46	E,E‐α‐farnesene	1,510	0.1
47	γ‐cadinene	1517	0.1
48	δ‐cadinene	1526	0.3
49	β‐thujaplicinol	1537	0.2
50	α‐calacorene	1547	0.2
51	E‐ρ‐methoxy cinnamaldehyde	1572	0.5
52	Spathulenol	1583	1.8
53	Caryophyllene oxide	1588	1.3
54	*n*‐hexadecane	1601	*T*
55	Ledol	1606	0.2
56	1,10‐di‐epi‐cubenol	1622	1.1
57	Epi‐α‐muurolol	1648	0.2
58	3‐thujopsanone	1655	0.2
59	2Z,6E‐farnesol	1,730	0.5
60	2E,6E‐farnesol	1748	0.6
61	Tetradecanoic acid	1,770	0.4
62	Neophytadiene	1842	0.6
63	Hexahydrofarnesyl acetone	1851	0.5
64	Methyl hexadecanoate	1925	0.1
65	Isophytol	1952	0.2
66	Dibutyl phthalate	1968	0.3
67	*n*‐hexadecanoic acid	1972	3.2
68	Eicosane	2001	0.1
**69**	***cis*‐phytol**	**2,122**	**9.7**
70	Ethyl linoleate	2,161	1.7
	**Major Compound Groups**		
	Monoterpene hydrocarbon		0.6
	Oxygenated monoterpene		55.4
	Sesquiterpen hydrocarbon		3.4
	Oxygenated sesquiterpene		6.7
	Diterpenoide		9.9
	Phenyl propanoides		16.7
	Miscellaneous		6.9
	**Total Identified**		**99**.**6**

Note

Major compounds are shown in bold.

^1^ RI: Retention Index on the HP‐5 MS column.

^2^ *t*: trace (<0.1%).

### 3.2 Total phenolic (TPC) and total flavonoid contents (TFC)

**FIGURE 1:**
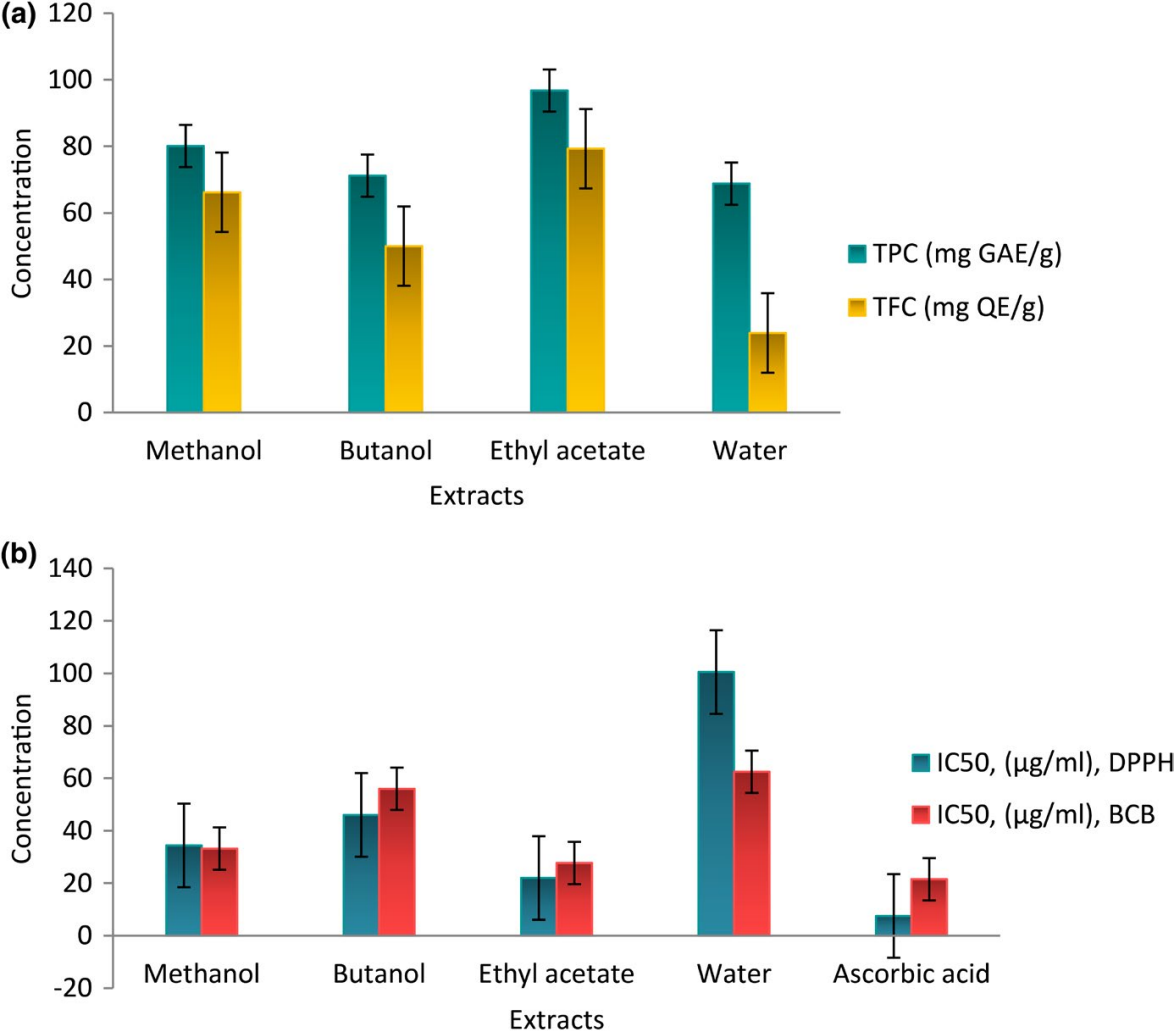
Total phenolic and total flavonoid contents (a) and antioxidant activities (b) of *Deracocephalum moldavica* extracts

The total phenolic content (TPC) of extracts from *D. moldavica* is most commonly estimated by the Folin‐Ciocalteu method. In this analytical method, phenolic compounds are deprotonated and form phenolate ions that react with the Folin–Ciocalteu reagent (phosphomolybdate and phosphotungstate), resulting in a blue color, which absorbs visible light with a maximum around 765 nm (Vazquez et al., 2015), while the method for the determination of TFC is based on the formation of flavonoid–aluminum complexes with a maximum absorbance at 410–430 nm (Pękal, 2014). TPC and TFC of the extracts are presented in Figure 1a in aqueous and EtOAc extracts, respectively. The highest TPC was determined in EtOAc extract (96.8 ± 1.5 mg GAE g^‐1^), followed by the MeOH extract, 80.1 ± 2.3 mg GAE g^‐1^. The lowest TPC was measured in the aqueous extract, 68.8 ± 2.4 mg GAE g^‐1^. TFC was in the range from 23.9 ± 1.2 (in aqueous extract) to 79.3 ± 2.5 mg QE g^‐1^ (in EtOAc extract). In the literature, the antioxidant activity and TPC of a 70% aqueous MeOH extract of *D. moldavica* was evaluated by (Weremczuk‐jeżyna et al., 2017). The TPC of the aerial parts of *D. moldavica* was 110.1 mg GAE g^‐1^, which was higher than observed in our study. In another study, by Aprotosoaiea et al. (2016), the TPC of the aerial parts of *D. moldavica* was 289.55 ± 2.63 mg of GAE g^‐1^, which was also higher than found for the MeOH extract in our study. Furthermore, Dastmalchi et al., (2007) observed a higher TPC for the 80% MeOH extract of the aerial parts of Iranian *D. moldavica* (488.4 ± 1.8 mg/g), but lower amounts for the EtOAc extracts compared to our samples.

### 3.3 Antioxidant activity and UPLC/ESI‐QTOF‐MS analysis

Among the extracts of *D. moldavica*, the EtOAc one exhibited the strongest scavenging activity with an IC_50_ value of 22.0 ± 2.1 µg.ml^‐1^ which is less active than ascorbic acid as positive control (IC_50_ = 7.5 ± 0.2 μg.ml^‐1^) (Figure 1b). Antioxidant activity was also found in the MeOH extract (IC_50_ = 34.4 ± 2.5 µg.ml^‐1^). The potent free radical scavenging activity of the MeOH extract of *D. moldavica* confirmed Dastmalchi et al., (2007), who revealed that the MeOH extract was a significantly better scavenger than quercetin. It is also in accordance with another study which reported scavenging effects of the MeOH extract of *D. moldavica* in the DPPH assay (EC_50_ = 23.10 ± 0.10 μg.ml^‐1^) (Aprotosoaie et al., 2016). In the BCB method, the EtOAc extract again exerted the strongest β‐carotene inhibition activity (94% inhibition, at 150 µg.ml^‐1^) followed by the MeOH (82%), n‐BuOH (75%), and aqueous (59%) extracts (Figure 1b). In the present study, UPLC/ESI‐QTOF‐MS was carried out on the extracts with the highest antioxidant activity to find the compounds potentially responsible for the antioxidant activity. The antioxidant activity of the MeOH and especially the EtOAc extracts of *D. moldavica* was in accordance with their amounts of phenolic acids. The UPLC/ESI‐QTOF‐MS analysis (Table 2) revealed that the MeOH extract of *D. moldavica* contains high amounts of phenolic acids, including rosmarinic acid (34,407 ± 694 µg.g^‐1^) and 2‐hydroxycinnamic acid (15,124 ± 2000 µg.g^‐1^), and of 4‐hydroxycoumarin (5,216 ± 95 µg.g^‐1^). In the literature, rosmarinic acid was also found to have the highest concentration in a MeOH extract of an Iranian *D. moldavica* (89,083 ± 1,380 μg.g^‐1^) (Dastmalchi et al., 2007). In our study, a much higher concentration of rosmarinic acid (75,508 ± 1,044 μg.g^‐1^) than in the MeOH extract was found in the EtOAc extract, followed by caffeic acid (69,678 ± 5,578 μg.g^‐1^), 3‐hydroxybenzoic acid (35,368 ± 2,803 μg.g^‐1^), and 2‐hydroxycinnamic acid (23,466 ± 2,122 μg.g^‐1^). It is evident from our results that the compounds most responsible for high antioxidant capacity of *D. moldavica* were phenolic acids such as rosmarinic acid, caffeic acid, hydroxycinnamic acids, and hydroxycinnamic acid. The antioxidant activity of rosmarinic acid, an ester of caffeic acid and 3,4‐dihydroxyphenyllactic acid, has already been demonstrated both in vitro and in vivo by many researchers (Adomako‐Bonsu et al., 2017; Nicolai et al., 2016; Tsai et al., 2019).

**TABLE 2:** Phenolic compounds quantified in the evaluated extracts from *Deracocephalum moldavica*, presented as mean ±standard deviation (μg.g^‐1^)

	Compounds	MeOH extract	EtOAC extract	Molecular formula	Molecular weight (M)	HPLC ESI‐MS (*m/z*)	
						RT (min)	[M‐H]^‐^
1	Malic acid	255 ± 59	73.7 ± 46.6	C4H6O5	134.087	0.94	133.014
2	Quinic acid	463 ± 29	72.3 ± 3.8	C7H12O6	192.167	0.96	191.120
3	Succinic acid	4,527 ± 902	5,072 ± 131.2	C4H6O4	118.088	1.21	117.018
4	Citric acid	5,101 ± 397	46.2 ± 3.8	C6H8O7	192.123	1.22	191.102
5	Pyrogallol	2.4 ± 0.7	11.75 ± 0.2	C6H6O3	126.111	1.24	125.024
6	Gallic acid	17 ± 1.2	79.4 ± 2.4	C7H6O5	170.022	1.33	168.90
7	Pyrocatechol	6.7 ± 0.04	141 ± 8	C6H6O2	110.112	1.95	109.028
8	3–4‐Hydroxybenzoic acid	56.7 ± 0.15	1,151 ± 62.7	C7H6O4	154.121	2.01	153.010
9	Catechin	0.76 ± 0.24	0.20 ± 0.03	C15H14O6	290.271	2.21	289.064
10	Chlorogenic acid	1,359 ± 100	288 ± 16	C16H18O9	354.311	2.37	353.202
11	4‐Hydroxybenzoic acid	70 ± 5.2	2,867 ± 240	C7H6O3	138.122	2.8	137.050
12	3‐Hydroxybenzoic acid	535 ± 486	35,368 ± 2,803	C7H6O3	138.122	2.83	137.025
13	Esculetin	31 ± 1.7	888.9 ± 0.52	C9H6O4	178.143	3.03	177.018
14	Vanillic acid	97.8 ± 29	755.5 ± 29.65	C8H8O4	168.148	3.13	167.036
15	Syringic acid	39 ± 1.9	107.4 ± 2.7	C9H10O5	198.174	3.17	197.045
16	Caffeic acid	3,019 ± 44	69,678 ± 5,578	C9H8O4	180.159	3.19	179.035
17	Epicatechin	0.33 ± 0.01	0.48 ± 0.28	C15H14O6	290.271	3.84	289.064
18	4‐Hydroxycinnamic acid	80 ± 3.6	1587 ± 80.8	C9H8O3	164.160	4.54	163.042
19	3‐Hydroxycinnamic acid	121 ± 10.6	2,146 ± 90	C9H8O3	164.160	4.56	163.042
20	Rutin	668 ± 8.8	530 ± 43.3	C27H30O16	610.153	4.71	609.1
21	Sinapic acid	0.96 ± 0.2	16.7 ± 1.10	C11H12O5	224.212	4.88	223.061
22	Ferulic acid	10 ± 7.0	416 ± 0.80	C10H10O4	194.186	5.05	193.050
23	2‐ Hydroxycinnamic acid	15,124 ± 2000	23,466 ± 2,122	C9H8O3	164.160	5.14	163.042
24	Tannic acid	4,069 ± 2,101	73.45 ± 11.50	C76H52O46	1701.206	5.31	1,700.080
25	Naringin	965 ± 17.7	11 ± 2.4	C27H32O14	580.539	5.84	579.173
26	Benzoic acid	662 ± 54.25	3,268 ± 20	C7H6O2	122.123	5.97	121.031
27	Quercitrin	26 ± 8.4	321.4 ± 42	C21H20O11	448.38	6.02	447.120
28	Hesperidin	1,030 ± 251	789.7 ± 513	C28H34O15	610.565	6.24	609.172
29	Rosmarinic acid	34,407 ± 694	75,508 ± 1,044	C18H16O8	360.318	6.94	359.054
30	4‐Hydroxycoumarin	5,216 ± 95	7,215 ± 158	C9H8O3	164.160	7.04	163.042
31	Salicylic acid	3.20 ± 0.10	20.92 ± 0.07	C7H6O3	138.122	7.38	137.025
32	Resveratrol acid	1.3 ± 0.04	53.13 ± 3.3	C14H12O3	228.247	8.24	227.072
33	Luteolin	5.6 ± 2.85	7.5 ± 0.2	C15H10O6	286.239	8.87	285.040
34	Quercitin	1.5 ± 0.12	12.4 ± 1.2	C15H10O7	302.238	9.11	301.000
35	Naringenin	33.4 ± 4.2	114.9 ± 0.6	C15H12O5	272.256	10.77	271.061
36	Hesperetin	9.9 ± 0.36	19.9 ± 9.8	C16H14O6	302.282	11.04	301.015
37	Kaempferol	38.7 ± 17.6	134 ± 4.5	C15H10O6	286.239	11.12	285.040

Abbreviation: ND, not detected.

### 3.4 Cytotoxic activity

Extracts of *D. moldavica* in a concentration range from 50 to 400 μg.ml^‐1^ were assayed for their cytotoxic activity against two human cancer cell lines, SW‐48 and MCF‐7, and against a normal cell line, NIH/3T3. None of the extracts (50–400 μg.ml^‐1^) exhibited cytotoxic activity, suggesting potential safety of the plant. This is in accordance with a study by Yu et al., (2019) who did not found a significant cytotoxic effect of the EtOAc extract of *D. moldavica* (33.3% growth inhibition at 100 μg.ml^‐1^) against human epidermal keratinocyte (HaCaT) cells. To the best of our knowledge, there is no other published data on the cytotoxicity of *D. moldavica* extracts.

## 4 CONCLUSION

The antioxidant and cytotoxic activities of different extracts of *D. moldavica*, that is, EtOAc, MeOH, n‐BuOH and aqueous extracts, the total phenolic and flavonoid contents as well as the phytochemical profiles of the EO and the extracts were determined. The EtOAc and MeOH extracts were found to possess remarkable antioxidant activity in the DPPH and BCB assays. GC‐MS analysis showed that the majority of the compounds in the EO were oxygenated monoterpenes (55.4%). Further, UPLC–QTOF–MS analysis allowed identifying 37 metabolites, mainly pertaining to phenolic acids. Rosmarinic acid occurs in high amounts in the EtOAc and MeOH extracts of *D. moldavica* and may be responsible for most of the antioxidant activity. Our UPLC/PDA‐MS analysis focused on the quantification of some specific phenolic compounds. Thus, further studies are required to identify other compounds that may be present in significant amounts, but were not determined. None of the extracts, even at high concentrations (400 μg.ml^‐1^), showed considerable cytotoxicity, which suggests potential safety of the plant to be used as a natural preservative in food.

## CONFLICT OF INTEREST

No conflict of interest was reported by the authors.

## AUTHOR CONTRIBUTION

**Azin Fattahi:** Investigation (equal). **Abolfazl Shakeri:** Conceptualization (equal); Investigation (equal); Writing‐original draft (lead). **Zahra Tayarani‐Najaran:** Software (equal). **Mourad Kharbach:** Methodology (equal). **Karen Segers:** Methodology (equal). **Yvan Vander Heyden:** Methodology (equal). **Seyedeh Faezeh Taghizadeh:** Formal analysis (equal); Software (equal). **Hanieh Rahmani:** Investigation (equal). **Javad Asili:** Conceptualization (equal); Funding acquisition (equal).
